# Compounds with capacity to quench the tyrosyl radical in *Pseudomonas aeruginosa* ribonucleotide reductase

**DOI:** 10.1007/s00775-019-01679-w

**Published:** 2019-06-20

**Authors:** Gustav Berggren, Margareta Sahlin, Mikael Crona, Fredrik Tholander, Britt-Marie Sjöberg

**Affiliations:** 1 Department of Chemistry, Ångström Laboratory, Uppsala University, Uppsala, Sweden; 2 Department of Biochemistry and Biophysics, Stockholm University, Stockholm, Sweden; 3 Swedish Orphan Biovitrum AB, Stockholm, Sweden

**Keywords:** Diferric-oxo center, Radicals, Inhibitors, Ribonucleotide reductase, Thermal shift analysis, EPR

## Abstract

Ribonucleotide reductase (RNR) has been extensively probed as a target enzyme in the search for selective antibiotics. Here we report on the mechanism of inhibition of nine compounds, serving as representative examples of three different inhibitor classes previously identified by us to efficiently inhibit RNR. The interaction between the inhibitors and *Pseudomonas aeruginosa* RNR was elucidated using a combination of electron paramagnetic resonance spectroscopy and thermal shift analysis. All nine inhibitors were found to efficiently quench the tyrosyl radical present in RNR, required for catalysis. Three different mechanisms of radical quenching were identified, and shown to depend on reduction potential of the assay solution and quaternary structure of the protein complex. These results form a good foundation for further development of *P. aeruginosa* selective antibiotics. Moreover, this study underscores the complex nature of RNR inhibition and the need for detailed spectroscopic studies to unravel the mechanism of RNR inhibitors.

## Introduction

Ribonucleotide reductase (RNR) catalyzes the conversion of ribonucleotides into deoxyribonucleotides, and represents the only de novo pathway for the synthesis of DNA building blocks [1–4]. Consequently, RNR is essential to all free-living organisms, making it a potential drug target in a wide variety of organisms. To date, three different classes of RNR are known, denoted class I, II, and III, respectively, each dependent on different cofactors [1, 5]. While bacteria can feature any combination of the three classes, eukaryotes are generally dependent on class I. Thus, any antibiotic targeting class I RNR has to display a high degree of selectivity. However, it is noteworthy that even when comparing class I RNR from human to different bacterial organisms the sequence identity is generally below 50%; thus, there is ample opportunity for designing species-specific RNR inhibitors.

In class I RNRs, the enzyme is heteromeric and built up by two different subunits denoted the α- and the β-subunit, respectively. The active form of the enzyme is considered to be the tetrameric complex (α_2_β_2_) [6, 7]. The reduction of ribonucleotides occurs in the α-subunit denoted NrdA, but the reaction is dependent on long-range transfer of an electron hole from a radical located in the β-subunit denoted NrdB [8–10]. In subclasses Ia and Ib, the radical is a stable tyrosyl radical in the vicinity of a *M*_2_^III,III^ metal site (*M* = Fe or Mn); in subclasses Ic and Id, it resides in a *M*^IV^–*M*^III^ metal site; and in subclass Ie, it resides on a 3,4-dihydroxyphenylalanine residue originating from a tyrosyl residue [5]. As a result, there are a number of potential targets for inhibitors, e.g., the active site in the α-subunit, the radical in the β-subunit, or interfering with oligomer formation via the allosteric site or by blocking protein–protein contact surfaces [4]. Unfortunately development of RNR inhibitors has historically been hampered by the labor-intensive nature of RNR enzymatic assays. Our report of a high-throughput method for discovering new antimicrobial RNR inhibitors dismissed this obstacle [11]. In the original proof-of-concept study, 1364 substances were evaluated for their capacity to inhibit the activity of class Ia RNR from the opportunistic pathogen *Pseudomonas aeruginosa* [11], a bacterium that encodes genes for all three classes of RNR [4].

Antibiotic resistance has grown during the last decade and *P. aeruginosa* is the sixth most common nosocomial pathogen in hospitalized patients and causes more than 50,000 infections per year in the USA healthcare system [12, 13]. Here we report a continuation of our initial screen for *P. aeruginosa* RNR inhibitors with an investigation of the inhibition mechanism of nine compounds previously discovered to inhibit the RNR activity by 90% or more [11]. The inhibitors were selected as representative examples of different structural subclasses of inhibitors, i.e., naphthoquinone-like or phenol-containing compounds, as well as a more diverse group of aromatic inhibitors many of which feature heterocyclic structural elements. The binding of the inhibitors to the α- and β-subunit was probed by thermal shift analysis (TSA). Electron paramagnetic resonance (EPR) spectroscopy was employed to study the influence of the inhibitors on the β-subunit, by monitoring their ability to quench the tyrosyl radical. Four of the compounds inhibited the β-subunit directly, two compounds inhibited the β-subunit only in the presence of a reducing agent, and three compounds inhibited the active holoenzyme complex. As several of the compounds have excellent standard solubility and permeability measures of drug and lead likeness, our study forms a good start for future development of lead compounds against *P. aeruginosa* RNR.

## Materials and methods

*General*—chemical reagents were purchased from Sigma-Aldrich and used as received unless otherwise stated. Protein purity was assessed by gel electrophoresis by loading samples on PhastGel™ Gradient 10–15 precast gels (GE Healthcare) with Precision Plus Protein™ standards (Bio-Rad). Protein concentrations were determined with the Bio-Rad Protein Assay, using bovine serum albumin as a standard and refer to dimeric proteins. NrdA2 and NrdB of class I RNR from *P. aeruginosa* were purified as previously described [14]. The inhibitors were obtained from the NCI/Development Therapeutics Program Open Chemical Repository (diversity set II) and used as received. Out of the 1364 compounds in the original set, 9 substances were included in this study as they had shown > 90% inhibition of *P. aeruginosa* RNR in our original screening study [11].

*Drug likeness analyses*—To provide an estimate on compound solubility and permeability, standard measures of drug and lead likeness passing the Lipinski/Ghose/Veber/Egan/Muegge filter were calculated using SwissADME [15].

*Thermal shift analyses*—TSA assays were performed using differential static light scattering on a Stargazer-384 (Harbinger Biotechnology and Engineering Corporation, Canada) instrument in 384-well optical bottom plates (Nunc, USA). The assay mixtures (50 µl) contained *P. aeruginosa* NrdA2 (2 µM) or NrdB (5 µM) in 50 mM HEPES pH 7.5, 5 mM TCEP, and 100 µM test compound dissolved in DMSO. For the NrdB mixtures, TCEP was omitted and 0.4 M Guanidine-HCl was included to unfold the protein within the applied temperature ramping range. After the final addition of compounds to be analyzed, the assay mixtures were covered with 40 µl mineral oil and plates centrifuged at 3000 rpm for 5 min in a plate centrifuge (Hettich Universal 320, Germany). Plates were heated at 1 °C/min in the range of 25–80 °C with images captured every 30 s. Using the provided software (Harbinger Biotechnology and Engineering Corporation), light scattering intensities from the images were plotted as a function of temperature and the aggregation temperature calculated. ∆*T* represents the difference between the aggregation temperature of the protein with (compound in DMSO) and without (only DMSO) the potential ligand. The data shown represent the mean and standard deviation of two samples unless otherwise indicated.

*Sample preparation for EPR studies*—three different setups of incubation with inhibitors were used to delineate the effects of inhibitors on *P. aeruginosa* RNR. In all experiments, the final concentration of DMSO, needed to keep the inhibitors in solution, was 1% and the concentration of inhibitor was 133 µM. Protein concentrations refer to the homodimeric subunits. (1) When testing the quenching effect on the tyrosyl radical in isolated NrdB, the protein concentration was 15 µM. Three equivalents of Fe^2+^ per NrdB in 50 mM Tris pH 7.5 were added to reconstitute the metal-radical site. After addition of the inhibitor compound, the mixture was flash frozen at different time points. For additional kinetic information, samples were thawed and frozen in several cycles. (2) When testing the effect of reduced inhibitors on NrdB, the protein concentration was 12 µM in 50 mM Tris pH 7.5. For each mixture, three equivalents of Fe^2+^ per NrdB were added, and after 10 s DTT (to a final concentration of 30 mM), after which the substance was added. Each mixture was immediately divided into two EPR tubes; the first was frozen directly and the second was frozen after 10-min incubation. All samples were prepared from the same stock of NrdB and the control was prepared without the presence of inhibitor. (3) When testing the effect of compounds on the active holoenzyme, the concentration of NrdA2 and freshly reconstituted NrdB was 12 µM in 50 mM Tris pH 7.5, 5 mM ATP, 10 mM magnesium acetate, and 30 mM DTT. After addition of the inhibitor compound, each mixture was immediately divided into two EPR tubes; the first was immediately frozen and the second was frozen after 2-min incubation. For further kinetics, the latter sample was thawed and frozen in several cycles. For all experiments, reference samples in the absence of inhibitor were prepared of the isolated NrdB protein ± DTT or the NrdA/B complex, and incubated under otherwise identical conditions. The radical decay rates for NrdB controls and NrdA/B controls were between 0.055 and 0.084 min^−1^, whereas NrdB + DTT controls showed no decay.

*EPR measurements*—EPR spectra were recorded on a Bruker Elexsys 500 series X-band spectrometer. Spectra at 77 K were recorded using a cold finger Dewar, and a liquid nitrogen flow system was used for temperatures at 100 K. Quantifications were made by comparing the double integral of the samples with that of a Cu^2+^/EDTA (1 mM/10 mM) standard under non-saturating conditions: microwave frequency 9.4 GHz, modulation amplitude 2 G, microwave power 1.5 mW.

## Results

### Chemical properties of the *P. aeruginosa* inhibitors

The chemical structure and drug likeness of the selected inhibitors are summarized in Table 1 together with their previously determined IC_50_ values for *P. aeruginosa* class I RNR. Four of the nine compounds are naphthoquinone-like, and two are phenol-containing. Five of the compounds have excellent drug likeness.

**Table 1:**
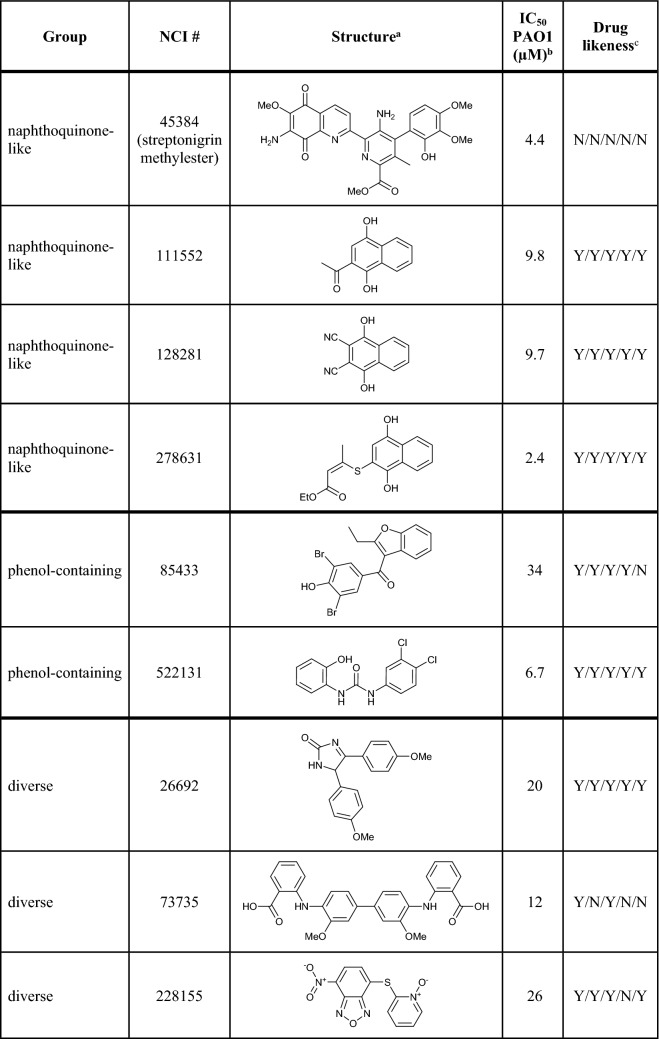
Substances in group order, formulas, *P. aeruginosa* class I RNR IC_50_ values, and drug likeness

^a^Structures obtained from the NCI database (Me: Methyl; Et: Ethyl)

^b^*In vitro* values obtained from [11]

^c^Passing the Lipinski/Ghose/Veber/Egan/Muegge filter. Calculated using SwissADME [15]

### Binding of the inhibitor to NrdA and NrdB

The binding of the inhibitors to the isolated *P. aeruginosa* NrdA and NrdB proteins was evaluated by TSA [16]. This method detects binding of small molecules to the protein as an increase in thermal stability. The TSA data are summarized in Table 2, and the observed temperature shifts clearly indicate that the two naphthoquinone-like compounds, NSC111552 and NSC128281, plus compounds NSC73735 and NSC228155, bind selectively to NrdA. NrdB-selective compounds are the naphthoquinone-like NSC45384 and the phenol-containing NSC522131. Additionally, the phenol-containing compound NSC85433 stabilizes both NrdA and NrdB. Conversely, the naphthoquinone-like compound NSC278631 and compound NSC26692 did not display any significant stabilization effect for either NrdA or NrdB. No clear trend was observed for the naphthoquinone-like compounds, while the affinity of both phenol-containing derivatives toward NrdB suggests that this motif is highly beneficial for NrdB binding.

**Table 2:** Temperature shifts after binding of substances to *P. aeruginosa* NrdA and NrdB

NCI#	Temperature shift ∆T (°C)^a^	
	NrdA	NrdB
45384	0.67 ± 0.64	**39.63**
111552	**1.39 ± 0.22**	− 6.92 ± 0.32
128281	**2.03 ± 0.76**	− 6.83 ± 0.08
278631	− 0.60 ± 1.09	− 5.82 ± 0.67
85433	**1.53 ± 0.38**	**19.03 ± 2.43**
522131	n.d.	**26.50 ± 3.24**
26692	0.39 ± 0.19	0.25
73735	**3.55 ± 0.74**	0.82 ± 0.12
228155	**5.13 ± 0.31**	− 7.43 ± 0.23

Mixtures of 2 µM of NrdA or 5 µM NrdB were treated as described in Materials and Methods

^a^*n.d.* not determined; values without standard deviation refer to one measurement; values indicating binding are shown in bold

### Quenching of the tyrosyl radical in NrdB

Arguably, most established RNR inhibitors act via quenching of the metal-tyrosyl radical site present in NrdB, either via electron transfer to the radical or by chelating the metals, thereby preventing the hole transfer to the active site in NrdA required to initiate the reaction. Consequently, the capacity of the inhibitors to reduce the tyrosyl radical present in *P. aeruginosa* NrdB was evaluated either in the presence or absence of a sacrificial electron donor (DTT). The EPR results are summarized in Table 3 together with the potential of the different compounds to become reduced by DTT employed in the enzymatic assays. The UV–Vis profiles on which the results in the rightmost column of Table 3 are based on are shown in Fig. S1 (Supplementary material).

**Table 3:** Tyrosyl radical decay in *P. aeruginosa* NrdB mediated by selected substances and their redox activity

NCI#	Tyrosyl radical decay rate and radical left after 10 min		Redox activity of compound^c^
	NrdB only^a^	plus 30 mM DTT^b^	
45384	No decay	0%	Yes
111552	0.19 ± 0.03 min^−1^ (20%)	20%	Yes
128281	0.30 ± 0.07 min^−1^ (20%)	80%	Yes
278631	0.40 ± 0.06 min^−1^ (10%)	0%	n.d.*
85433	No decay	No decay	n.d.
522131	0.35 ± 0.04 min^−1^ (20%)	20%	Yes
26692	No decay	No decay	Yes
73735	No decay	No decay	n.d.*
228155	No decay	10%	Yes

Mixtures with NrdB were incubated with 133 µM substance as described in Materials and Methods. All decay rates are corrected for decay of the NrdB radical in absence of inhibitor compound

*n.d.* no difference detected, * compounds with minor changes in UV–Vis profile

^a^Decay rates are shown, and within parenthesis radical left after 10 min

^b^Tyrosyl radical left after 10 min is shown

^c^Differences in UV–Vis profile of test compounds ± 6 mM DTT (see Fig. S1, Supplementary material)

**Fig. 1:**
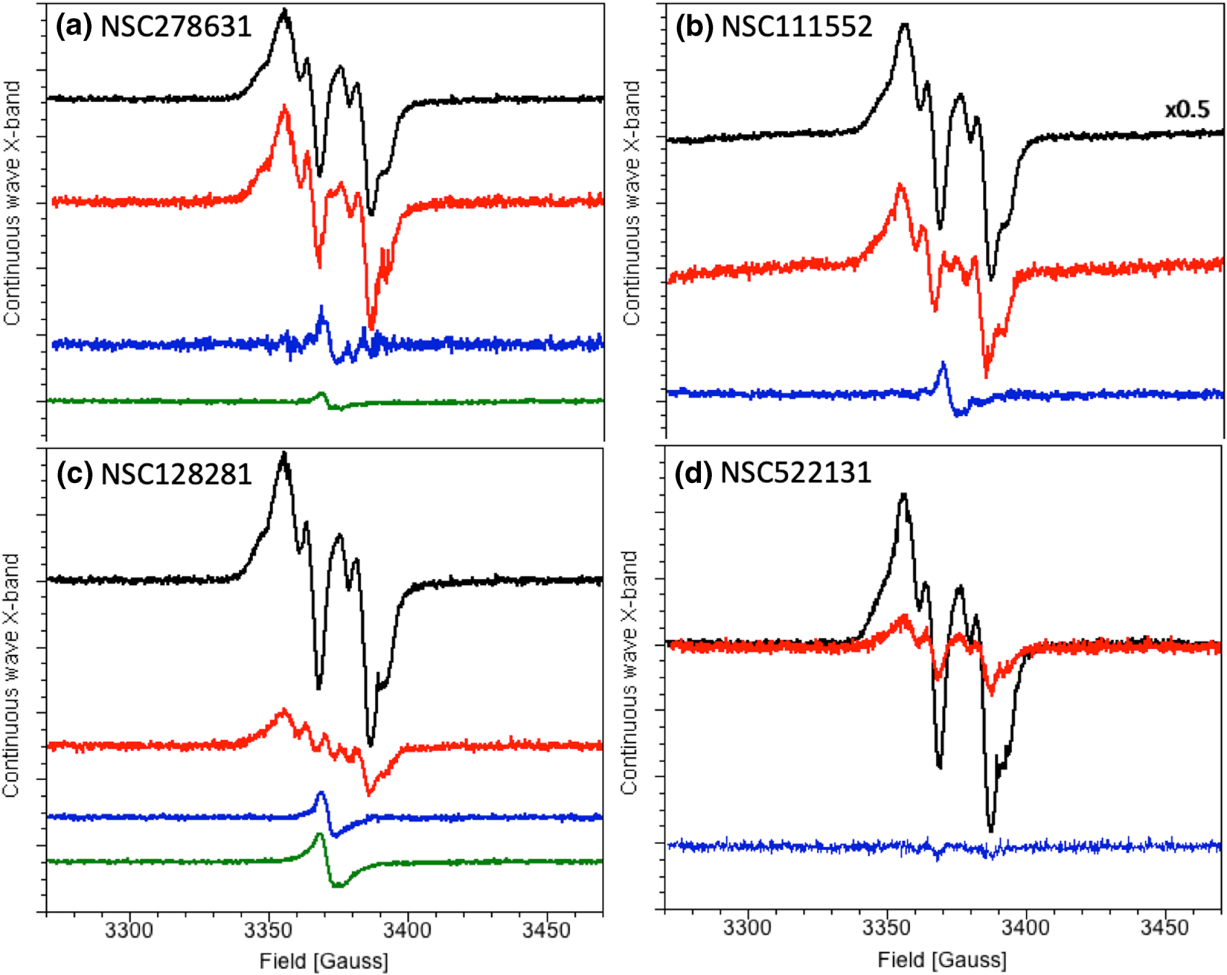
Decay of *P. aeruginosa* NrdB tyrosyl radical in presence of NSC compounds. **a** NrdB control after 60 s (*black*), NrdB plus substance NSC278631 incubated in buffer for 60 s (*red*), remaining substance-specific EPR signal after subtraction of NrdB-specific part (*blue*), and radical signal of only substance NSC278631 incubated in buffer (*green*). **b** NrdB control after 60 s multiplied by 0.5 for better illustration (*black*), NrdB plus substance NSC111552 incubated in buffer for 10 min (*red*), and remaining substance-specific EPR signal after subtraction of NrdB-specific part (*blue*). **c** NrdB control after 60 s (*black*), NrdB plus substance NSC128281 incubated in buffer for 10 min (*red*), remaining substance-specific EPR signal after subtraction of NrdB-specific part (*blue*) and radical signal of only substance NSC128281 incubated in buffer (*green*). **d** NrdB control after 60 s incubation (*black*), NrdB plus substance NSC522131 incubated in buffer for 10 min (*red*). All samples contained NrdB (15 µM) in 50 mM Tris pH 7.5 with 1% DMSO and 133 µM inhibitor substance. Spectra were recorded at 9.4 GHz, 77 K, modulation amplitude 2G, microwave power 1.5 mW, 4 scans

In our standard enzymatic assays, the radical generating Fe_2_^III,III^ -Y• site in NrdB is reconstituted by adding Fe^2+^ ions to apoNrdB immediately prior to the assay. This radical species features a distinct signal in EPR that allows us to probe its formation and rate of disappearance by X-band EPR spectroscopy. Four of the nine inhibitors studied, i.e., NSC111552, NCS128281, NSC278631, and NSC522131, quenched the radical in NrdB efficiently on the minutes time scale (Table 3), suggesting that these compounds are reducing enough to quench the radical even under aerobic, non-reducing conditions. In agreement with this, three of these substances, i.e., NCS278631, NCS111552, and NSC128281 also displayed new substance-specific EPR signals following their incubation with NrdB (*blue* spectra in Fig. 1a–c). The observed *g* values and width of these signals support their assignment as organic radicals; and in the case of NSC278631 and NSC128281, this signal was also clearly discernable for the isolated compounds in aqueous buffer (Fig. 1a, c, *green* spectra). This observation underscores that these compounds are at least semi-stable in their one-electron oxidized radical form, and shows that these naphthoquinone-like inhibitors are partially oxidized already upon solvation. Moreover, NSC278631 induced changes in the spectral shape of the remaining tyrosyl signal (Fig. 1a, *blue* spectrum). The latter is attributable to either a direct interaction between the inhibitor and the metal site, or alternatively, the binding of the inhibitor to another part of the protein resulting in structural changes around the radical. As NSC278631 did not display a positive shift in TSA (Table 2), this interaction conceivably leads to a destabilization of the protein. The fourth compound (NSC522131) did not reveal any new signal in the EPR spectra during quenching of the tyrosyl radical (Fig. 1d). The absence of a substance-specific signal in this latter case suggests that NCS522131 either dimerizes or decomposes further after electron transfer to NrdB.

It should be noted that DTT is usually added as a sacrificial electron donor to ensure enzyme turnover in RNR assays, and to facilitate continuous reactivation of the Fe_2_^III,III^-Y• cofactor. As many of the inhibitors outlined in Table 1 are potentially redox active, the effect of this reductant on the substances was probed by treating them with DTT and monitoring spectral changes in the UV–Vis spectrum (see Supplementary material Figure S1). As summarized in Table 3, six of the compounds, including three of the compounds that quenched the tyrosyl radical in NrdB, showed significant spectral changes in UV–Vis upon reduction in the presence of DTT. Two additional substances displayed small, but discernable, changes in their absorbance spectra, NCS73735 and NCS278631. The effect of the reductant on the radical quenching reaction was evaluated in a separate assay, in which EPR samples were prepared as described above but with DTT added to the reaction mixture after Fe^2+^ addition and before addition of inhibitor. Under these reducing conditions, two additional substances were found to completely quench (≥ 90%) the tyrosyl radical within 10 min, i.e., NCS45384 and NCS228155 (Table 3). This observation clearly supports the notion that they have reduction potentials in a range relevant for mediating electron transfer from the reductant to the Fe_2_^III,III^-Y• cofactor, resulting in the reduction of the tyrosyl radical. However, they could potentially inhibit the enzyme in an indirect fashion (see Discussion). In summary, a majority of the tested substances, including all naphthoquinone-like compounds, are clearly capable of quenching the tyrosyl radical in isolated samples of NrdB.

### Quenching of the tyrosyl radical in the holoenzyme

Finally, the five compounds that bound to NrdA were assayed with the complete enzymatic assembly. Under catalytically relevant conditions, the radical can be intercepted during hole transfer from NrdB to NrdA, or quenched in the active site located in NrdA. Thus, EPR samples were prepared containing enzymatically active mixtures of NrdA and NrdB. We also included NSC26692 in this test, since it was negative in all previous tests. Interestingly, all six tested substances quenched the tyrosyl radical during holoenzyme conditions (Table 4). Fastest quenching was promoted by NSC73735 and NSC228155, followed by NSC111552, NSC128281, and NSC85433 with half the rate, and NSC26692 with four times slower decay. The three compounds to which the radical in isolated NrdB was resistant (NSC85433 that binds both NrdA and NrdB, NSC73735 that binds only NrdA, and NSC26692 that did not bind to any subunit), all showed radical decay on the minutes time scale in the holoenzyme.

**Table 4:** Tyrosyl radical decay in *P. aeruginosa* RNR mediated by selected substances

NCI#	Tyrosyl radical decay rate (min^−1^)
111552	0.43 ± 0.32
128281	0.43 ± 0.03
85433	0.33 ± 0.03
26692	0.18 ± 0.19
73735	0.67 ± 0.01
228155	0.75 ± 0.36

Mixtures contained 12 µM each of NrdA and NrdB plus 5 mM ATP, 30 mM DTT, and 133 µM substance, and were treated as described in Materials and Methods. Decay rates are corrected for tyrosyl radical decay in absence of inhibitor

Thus, all nine inhibitors were found to quench the tyrosyl radical in NrdB, providing a first insight into their method of inhibition. However, the conditions of radical quenching varied significantly, showing mechanistic differences between the compounds. Of the two compounds that bound specifically to NrdB, only NSC522131 quenched the radical in the NrdB protein directly, whereas NSC45384 needed addition of the reductant DTT to quench the radical. Of the four compounds that bound specifically to NrdA, we were surprised to note that both NSC111552 and NSC128281 had the capacity to directly quench the radical in NrdB protein, whereas NSC228155 needed addition of the reductant DTT to quench the radical. These results suggest that they may inhibit the enzyme via more than one mechanism. Only NSC73735 behaved as expected for a compound with specificity for NrdA and quenched the radical only when presented to the active holoenzyme complex. Likewise, the compound that bound to both NrdA and NrdB only quenched the radical in the active holoenzyme. Finally, of the two compounds that lacked binding to both NrdA and NrdB in the TSA test, NSC278631 still had capacity to quench the radical in the isolated NrdB protein and NSC26692 quenched the radical in the active holoenzyme.

## Discussion

Our study has identified compounds that inhibit *P. aeruginosa* class I RNR by quenching the tyrosyl radical in three different ways. Four compounds quench the radical in the isolated NrdB protein, while three compounds quench the radical only in the active holoenzyme complex. Finally, two compounds quench the NrdB radical only under reducing conditions.

In related recent studies, two of the radical quenching compounds studied here have been reported to promote other redox-related reactions. NSC85433 is also metabolized by cytochrome P450 [17] and clinically used in treatment of gout where it appears to scavenge superoxide radicals [18]. Naphtho-1,4-quinones, like NSC111552, are reduced to hydro-1,4-quinones by many reducing agents and are readily oxidized again by air [19]. In addition, eight of the tested compounds are inactive in a majority of anticancer tests in human cells (Table S1, Supplementary material), suggesting that they are feasible starting compounds for developing selective antibiotics.

In the context of RNR inhibition, compounds NSC228155 and streptonigrin (the non-esterified version of NSC45384), were in an earlier study found to inhibit growth of *P. aeruginosa* as effectively as the common antibiotics tetracycline and carbenicillin [11]. In this study, we show that a major inhibitory mechanism of both NSC228155 and NSC45384 is efficient quenching of the radical in NrdB by the reduced compounds. However, their need for reducing conditions raises the possibility of an indirect inhibition rather than direct electron transfer to the radical site. Such indirect quenching can originate from either the catalytic formation of deleterious reactive oxygen species (ROS) or by chelating the Fe ions, thus preventing regeneration of the Fe_2_^III,III^-Y• cofactor following its spontaneous decay. One of these compounds, NSC228155, has recently been reported to generate hydrogen peroxide and superoxide radical and to promote protein dimerization [20–22], and a similar ROS-dependent inhibition cannot be ruled out here for *P. aeruginosa* class Ia RNR. Still, considering the improved thermal stability of the NrdA and NrdB protein induced by NSC45384 and NSC228155, respectively, it is unlikely that the inhibition mechanism is limited to ROS-induced effects. Conversely, and similarly to what was earlier observed for human RNR [23], we found that several of the compounds rather conferred decreased thermal stability to *P. aeruginosa* NrdB. In our study, four of the six compounds that quench the tyrosyl radical in the NrdB protein also decreased the thermal stability of the protein. This might be explained as oligomerization of the inhibited subunit or loss of cofactor, but unspecific binding cannot be ruled out. Moreover, four of the compounds studied here have earlier been tested as potential inhibitors of human RNR, but only NSC73735 showed a positive binding to human NrdA, and was found to lower all dNTP pools in HL-60 cells [23]. In our hands, NSC73735 increases the thermal stability of *P. aeruginosa* NrdA and quenches the NrdB radical in the active holoenzyme complex. This implies that NSC73735 may have a complex inhibition mechanism, and perhaps can bind to NrdA proteins from a variety of organisms.

In closing, it is interesting to note that the TSA test, which has been extensively used in drug screening campaigns [24, 25] indicated that three compounds bound specifically to NrdA, two bound specifically to NrdB, and one compound to both NrdA and NrdB. Still, these binding assays had only minor correlation with the radical quenching results observed in this study. Conceivably, the inhibitory mechanisms are complex for several of the compounds tested, and our EPR data underscore the need for more detailed spectroscopic studies to unravel the mechanism of RNR inhibitors.

## Abbreviations

apoNrdB: NrdB lacking metal-radical site
DMSO: Dimethyl sulfoxide
DTT: Dithiothreitol
HEPES: 2-[4-(2-hydroxyethyl)piperazin-1-yl]ethanesulfonic acid
RNR: Ribonucleotide reductase
TCEP: Tris(2-carboxyethyl)phosphine
TSA: Thermal shift assay
